# Source-Dependent Renal Effects of Commercial Protein Supplements: A 28-Day Study in Rats

**DOI:** 10.3390/medicina62101882

**Published:** 2026-09-29

**Authors:** Engin Sümer, Elif Nur Sarıalioğlu, Ayşe Aksan, Baraa Hamze, Aysun Özbay Önal, Muhammed Hamitoğlu, Ahmet Aydın

**Affiliations:** 1 Department of Experimental Research Unit, Faculty of Medicine, Yeditepe University, İstanbul 34755, Türkiye; 2 Department of Pharmaceutical Toxicology, Faculty of Pharmacy, Yeditepe University, İstanbul 34755, Türkiye; sarialiogluelifnur@gmail.com (E.N.S.); ayseaksan2001@hotmail.com (A.A.); mohammed.saz@yeditepe.edu.tr (M.H.); 3 Department of Clinical Pharmacy, Faculty of Pharmacy, Yeditepe University, İstanbul 34755, Türkiye; 4 Department of Histology and Embryology, Faculty of Medicine, İstanbul Aydın University, İstanbul 34295, Türkiye; aysunonal@aydin.edu.tr

**Keywords:** protein powder, kidney, renal histopathology, oxidative stress, casein, beef protein, creatinine, urea

## Abstract

*Background and Objectives*: Commercial protein powders are widely consumed to increase dietary protein intake, yet whether their renal effects differ by protein source remains poorly characterized. The primary objective of this study was therefore to determine whether 28 days of administration of commercial whey, casein, soy or beef protein powder produces source-dependent renal histopathological injury in rats, with the total renal injury score as the primary outcome. Secondary objectives were to assess conventional renal function markers and the renal oxidative stress response. *Materials and Methods*: Thirty-five female Sprague Dawley rats were stratified by body weight and randomly assigned to five groups (*n* = 7 per group): control, whey, casein, soy and beef. The powders were given by oral gavage in 0.9% sterile isotonic saline at 400 mg/kg/day of protein for 28 consecutive days; controls received the vehicle only. Body weight, kidney-to-body weight ratio, serum urea and creatinine, urinalysis, renal histopathology, renal oxidative stress parameters and the elemental composition of the powders were assessed. *Results*: Protein supplementation did not significantly affect body weight, kidney-to-body weight ratio, serum urea, creatinine or urinalysis findings in any group. Despite this, renal responses differed by protein source. All powders produced mild-to-moderate tubular change relative to the control. The beef group showed the highest total injury score and was the only group that differed from control in every parameter, although the protein groups did not differ from one another. The oxidative response also differed by source: renal malondialdehyde (MDA) was highest in the beef group and significantly exceeded the casein and soy groups, whereas catalase (CAT) activity was significantly reduced in the whey and casein groups. Glutathione peroxidase (GSH-Px) and nitrite levels were unchanged. Estimated metal exposure from all powders remained below toxicological risk thresholds. *Conclusions*: The renal effects of commercial protein powders are source-dependent even at a low dose and in the absence of any change in conventional renal function markers. Beef protein was associated with the highest lipid peroxidation and casein with the largest fall in enzymatic antioxidant capacity, two distinct oxidative signatures. These effects, and their progression under longer-term exposure, warrant further investigation.

## 1. Introduction

Commercial protein supplements are widely used by athletes, physically active individuals, and people seeking to increase their dietary protein intake or improve body composition [1]. The most commonly consumed products are the animal-derived proteins whey, casein and beef, and the plant-derived protein soy. Although all provide concentrated dietary protein and can comparably support gains in lean mass, these sources differ in amino acid composition, digestion kinetics, and non-protein constituents [2,3], and such differences may influence how the kidney responds to each.

Adequate dietary protein is essential for growth, tissue maintenance, and metabolic homeostasis; however, sustained high protein intake increases the renal workload and promotes glomerular hyperfiltration and intraglomerular hypertension [4]. In healthy individuals this response is generally well tolerated: systematic reviews and meta-analyses report no adverse effect of higher protein intake on renal function in adults without kidney disease [5,6,7]. In individuals with reduced renal reserve or pre-existing chronic kidney disease (CKD), however, sustained hyperfiltration has been implicated as a factor that may accelerate functional decline, and moderating animal-protein intake can reduce hyperfiltration and proteinuria [4]. Consistent with this, current guidelines recommend avoiding high protein intake (>1.3 g/kg/day) and favoring plant-predominant sources in patients at risk of progression [8]. Protein supplements are particularly relevant in this context because they deliver concentrated protein loads that can substantially exceed habitual dietary intake, yet whether different supplement sources differ in their renal impact remains poorly defined.

Importantly, the renal effects of protein intake may depend not only on the total amount consumed but also on the source and composition of the protein. Animal- and plant-derived proteins differ in amino acid profile, dietary acid load, and metabolic handling, and these differences may influence renal hemodynamics, oxidative stress, and tissue-level outcomes [2,4]. Because these compositional properties determine the acid load, mineral content and amino acid profile delivered to the kidney, the renal response to a protein supplement may depend on its source and not only on the amount ingested. Direct experimental support exists: in rats, substituting soy protein for casein at an equal protein intake slowed the progression of renal disease [9], and red meat protein raises lipid-peroxidation biomarkers through its heme iron content [10]. Comparing commercial protein powders from different sources under identical experimental conditions is therefore necessary to determine whether specific sources carry a greater renal risk than others.

Because serum urea and creatinine are relatively insensitive to early or localized tubular injury [11], a comparison between protein sources based on these markers alone could miss tissue-level effects. We therefore combined them with renal histopathology and with markers of lipid peroxidation and antioxidant capacity, which together detect structural and redox changes that precede overt functional loss [12].

Despite the widespread use of protein powders, experimental studies directly comparing different protein sources with respect to conventional renal markers, histopathology, and oxidative stress remain limited. We hypothesized that renal responses would differ according to protein source, with certain sources producing greater tissue-level and oxidative alterations than others, even in the absence of changes in conventional renal function markers. The primary objective of this study was therefore to determine whether 28 days of oral administration of commercial whey, casein, soy or beef protein powder, at a matched protein dose of 400 mg/kg/day, produces source-dependent renal histopathological injury in healthy young female Sprague Dawley rats, with the semi-quantitative total renal injury score as the primary outcome. Secondary objectives were to assess conventional renal function markers (serum urea and creatinine, kidney-to-body weight ratio and urinalysis) and the renal oxidative stress response (lipid peroxidation, enzymatic antioxidant activity and plasma nitrite). The elemental composition of the powders was analyzed to determine whether any observed effect could be attributed to metal exposure.

## 2. Materials and Methods

### 2.1. Selection and Preparation of Protein Supplements Derived from Various Sources

Commercial protein supplements derived from different protein sources were used in the present study, comprising whey (Türkiye), casein (USA), soy (Türkiye), and beef (Türkiye) protein powders. The declared nutritional composition of each supplement, including protein content and other listed constituents, is presented in Table 1.

For oral administration, each protein powder was freshly suspended in 3 mL of 0.9% sterile isotonic saline immediately before use and administered daily by oral gavage, while the control group received an equal volume of the vehicle. The protein dose was set at approximately 400 mg/kg of actual protein for each group. This dose was selected on the basis of previously published rodent studies investigating the physiological and renal effects of protein powder supplementation and falls within the biologically relevant range reported in the literature [13,14].

### 2.2. Experimental Animals and the Oral Administration Protocol

A total of 35 female Sprague Dawley rats (218 ± 13 g, 9–10 weeks old) were bred and housed at the Yeditepe University Experimental Research Unit (İstanbul, Türkiye). All procedures were performed in accordance with Directive 2010/63/EU and are reported in accordance with the ARRIVE 2.0 guidelines [15]. The study was approved by the Yeditepe University Animal Experiments Local Ethics Committee (approval no. 2024/05-04, approved on 5 April 2024). The study protocol was not prospectively registered. Animals were maintained under good laboratory practice (GLP) conditions (12:12 h light/dark cycle, 22 ± 2 °C, 50 ± 20% humidity), with ad libitum access to standard pelleted chow (Carfil, Torhout, Belgium) and municipal tap water. This water is analyzed periodically, complies with national drinking-water standards and was identical for all five groups. Animals were group-housed (three rats per cage) with nesting material and shelters as environmental enrichment. For randomization, baseline body weights were recorded and the animals were stratified by body weight into five groups (*n* = 7 per group), which were then randomly assigned to the experimental conditions. The five groups were control (vehicle only), whey, casein, soy, and beef protein powder, each administered at 400 mg/kg/day for 28 days (*n* = 7 per group). Predefined humane endpoints (>20% body-weight loss or signs of severe distress or immobility) were applied throughout the study, and no animal reached these criteria. At the end of the 28-day administration period, animals were anesthetized with a single intraperitoneal injection of ketamine hydrochloride (Ketasol^®^, VetViva Richter GmbH, Wels, Austria; 100 mg/kg) combined with xylazine (Rompun^®^, Bayer, Leverkusen, Germany; 8 mg/kg). Depth of anesthesia was confirmed by loss of the pedal withdrawal reflex. Animals were then euthanized by exsanguination through intracardiac puncture, which simultaneously provided the terminal blood sample (approximately 8 mL); death was confirmed by cessation of the heartbeat before the kidneys were removed.

Each powder was administered once daily at a consistent time of day through a sterile gavage needle (Instech FTP1875; Fisher Scientific, Waltham, MA, USA); control animals received the same volume of vehicle by the same route. Body weight was recorded at baseline and monitored weekly throughout the experimental period, with final body weights recorded before euthanasia; the weekly measurements were used to adjust the administered dose.

### 2.3. Urinalysis

On days 26 and 27, animals were individually housed in metabolic cages (Tecniplast, Buguggiate VA, Italy) for 24-h urine collection, with free access to water but not food during the collection period. Urine was analyzed using 10-parameter reagent strips (Laboquick Urine Strip; Köroğlu Medikal, Bolu, Türkiye) according to the manufacturer’s instructions and read at the manufacturer-specified reaction time. Of the ten parameters, urinary protein, pH, and ketones were used as screening indicators of renal handling, acid-base status, and metabolic state, respectively. Reagent-strip urinalysis was included as a rapid, low-cost screening step intended to detect gross abnormalities, namely frank proteinuria, hematuria, glucosuria or a marked shift in urinary pH, that would have indicated overt renal injury and prompted further quantitative investigation. Because semi-quantitative strip reading is not appropriate for between-group comparison, these data were neither analyzed statistically nor used to support any conclusion. Quantitative measurement of urinary protein, albumin and creatinine is recommended for future work.

### 2.4. Evaluation of Renal Function Markers

Blood collected at euthanasia (Section 2.2) was centrifuged at 1400× *g* for 10 min at 4 °C using a refrigerated centrifuge, and the separated serum was used to assess renal function markers. Serum urea and creatinine were measured on a BioSystems BA200 automated analyzer (BioSystems, Barcelona, Spain); creatinine was determined by the Jaffé-compensated method and urea by the urease–glutamate dehydrogenase (GLDH) method. Both assays were performed according to the manufacturer’s application protocols for the analyzer. Serum urea and creatinine served as conventional indicators of renal function.

### 2.5. Kidney Collection and Kidney-to-Body Weight Ratio

Following blood collection, the abdominal cavity was opened and both kidneys were excised and immediately weighed. The kidney-to-body weight ratio was calculated from the combined weight of both kidneys as total kidney weight (g) × 100/body weight (g). This parameter was used to evaluate whether protein powder administration induced gross renal hypertrophy or atrophy at the organ-weight level.

### 2.6. Histopathological Evaluation of Kidney Tissue

One kidney from each rat was fixed in 10% neutral buffered formalin for histological analysis. The tissue underwent routine histological processing, including dehydration through a graded ethanol series, clearing in xylene (Sigma-Aldrich, St. Louis, MO, USA), and embedding in paraffin. Sections (5 μm thick) were cut from the paraffin blocks and stained with hematoxylin and eosin (H&E). Tubular dilation, tubular desquamation, tubular vacuolization and intertubular vascular congestion were selected as scoring parameters because they are established indicators of renal tissue injury in experimental dietary studies [12]. Sections were coded and scored by a single histologist blinded to group allocation. Each parameter was graded for every animal on an ordinal scale (0 = absent, 1 = mild, 2 = moderate, 3 = severe). A total renal injury score (range 0–12) was then calculated per animal as the sum of the four parameter scores.

### 2.7. Preparation of Kidney Homogenates for Oxidative Stress Analysis

Kidney tissues were used to assess oxidative stress-related parameters. For biochemical analysis, kidney samples were weighed and homogenized in a 1.15% KCl solution (4 mL per 1 g of tissue). The tissues were cut into small pieces in a Petri dish, the pre-measured KCl solution was added, and the mixtures were homogenized using a high-speed homogenizer under cold conditions. The homogenates were kept refrigerated during processing and then centrifuged at 2490× *g* for 10 min at 7 °C. The supernatants were collected, aliquoted into microcentrifuge tubes, and stored at −80 °C until further biochemical analysis.

### 2.8. Assessment of Oxidative Stress-Related Parameters in Kidney Homogenates

Oxidative stress-related parameters were measured spectrophotometrically using a Thermo Scientific Evolution 300 UV–Vis spectrophotometer (Thermo Fisher Scientific, Madison, WI, USA). Malondialdehyde (MDA) levels and catalase (CAT) and glutathione peroxidase (GSH-Px) activities were assessed in kidney tissue homogenates as indicators of lipid peroxidation and antioxidant defense, and nitrite levels were measured in plasma as an indirect marker of nitric oxide metabolism. MDA was measured as an index of lipid peroxidation, and CAT and GSH-Px as the principal enzymatic antioxidant defenses of renal tissue. The kidney is metabolically active and particularly vulnerable to reactive oxygen species, and these markers together reflect the balance between oxidative damage and antioxidant capacity. Protein concentrations in the kidney homogenates were determined using the Lowry method [16], with bovine serum albumin as the standard, and all oxidative stress parameters were normalized to these protein concentrations.

#### 2.8.1. Measurement of MDA Level

MDA levels were quantified using the thiobarbituric acid reactive substances assay. Kidney tissue homogenates were reacted with sodium dodecyl sulfate, acetic acid (pH 3.5), and thiobarbituric acid reagent, followed by incubation at 95 °C for 1 h. After cooling, the samples were centrifuged at 3150× *g* for 10 min, and the absorbance of the supernatant was measured at 532 nm. MDA concentrations were calculated using a standard curve prepared from tetramethoxypropane and normalized to the protein content of the kidney homogenate [17].

#### 2.8.2. Measurement of CAT Activity

CAT activity was determined according to Aebi’s method [18], based on the rate of hydrogen peroxide decomposition. Briefly, 1 mL of 10 mM hydrogen peroxide was added to 2 mL of kidney homogenate, and the decrease in absorbance was recorded at 240 nm at 30-s intervals. CAT activity was calculated from the change in absorbance and normalized to the protein content of the kidney homogenate.

#### 2.8.3. Measurement of GSH-Px Activity

GSH-Px activity was measured spectrophotometrically by monitoring the oxidation of NADPH at 340 nm. A reaction mixture containing Tris buffer, ethylenediaminetetraacetic acid, nicotinamide adenine dinucleotide phosphate, sodium azide, reduced glutathione, and glutathione reductase was prepared. For the assay, 10 µL of diluted kidney homogenate was added to 990 µL of the reaction mixture, and the baseline absorbance was recorded. The reaction was initiated by adding 10 µL of tert-butyl hydroperoxide, and after a 5-min incubation at room temperature, the absorbance was measured again [19]. GSH-Px activity was calculated from the rate of NADPH oxidation and normalized to the protein content of the kidney homogenate.

#### 2.8.4. Measurement of Plasma Nitrite Levels

Plasma nitrite concentrations were measured using the Griess reagent method (Thermo Fisher Scientific, USA). The assay is based on a diazotization reaction that produces a pink azo compound, the absorbance of which was measured at 540 nm [20].

### 2.9. Metal Element Levels in Protein Powders

Elemental analysis was performed to determine whether the tested protein powders contained metal levels that could represent a toxicological concern. For element analysis, the protein powders were digested in a microwave acid digestion system. Briefly, 5 mg of each powder was transferred into a Falcon tube, and 0.1 mL of hydrogen peroxide and 8 mL of nitric acid were added. The samples were then digested in a Multiwave GO Plus system (Anton Paar, Graz, Austria). Element levels in the digested samples were analyzed by inductively coupled plasma mass spectrometry (ICP-MS) using a Thermo Scientific iCAP Q series instrument (Bremen, Germany), applying the manufacturer’s recommended operating parameters. These results were used for the safety assessment of the protein powders. The Health Risk Index (HRI) was calculated as the estimated daily intake of each element divided by its oral reference dose [21], with values below 1 considered safe. The Tolerable Reference Value (TRV) for cadmium was based on the tolerable weekly intake of 2.5 µg/kg body weight established by the European Food Safety Authority, equivalent to 0.36 µg/kg body weight per day [22]. The Intake Reference Level (IRL) for lead was the interim reference level of 8.8 µg/day set by the United States Food and Drug Administration for females of childbearing age [23]. All calculations assumed a daily intake of 25 g of protein powder, corresponding to a typical single serving of the products tested (declared servings 25–35 g; Table 1). All measurements were performed in triplicate, and mean values are reported in Table 2.

### 2.10. Statistical Analysis

An a priori power analysis was conducted using G*Power 3.1.9.4 [24] to determine the required sample size. As the aim of this study was to assess the renal effects of the tested protein powders, renal histopathological injury was designated as the primary outcome and used as the basis for this calculation. For a fixed-effects one-way ANOVA across five groups (α = 0.05, power = 0.80) and assuming a large effect (Cohen’s f = 0.65) [9,12], a minimum of 35 animals (seven per group) was required, corresponding to an actual power of 0.825. Data are presented as mean ± standard deviation (SD). Normality was assessed using the Shapiro–Wilk test, and homogeneity of variance using the Brown–Forsythe test. Effect sizes for one-way ANOVA are reported as eta-squared (η^2^), equivalent to the R^2^ computed by the software. Normally distributed variables were analyzed using one-way or two-way ANOVA followed by Bonferroni’s post hoc test, whereas non-normally distributed variables were analyzed using the Kruskal–Wallis test followed by Dunn’s post hoc test. Ordinal histopathological scores were compared between groups using the Kruskal–Wallis test followed by Dunn’s multiple comparison test and are presented as median (minimum–maximum); the total renal injury score was the primary outcome. Body weight, which was measured repeatedly, was analyzed by two-way ANOVA (group × time), and all other parameters by one-way ANOVA. The sample size was determined for the primary histopathological outcome, and the study was therefore not powered to detect small differences in the secondary biochemical endpoints. Non-significant findings for these parameters should be interpreted as inconclusive rather than as evidence of no effect, and effect sizes are reported throughout to support this distinction. All analyses were performed using GraphPad Prism 10.5.0 (GraphPad Software, San Diego, CA, USA), and *p* < 0.05 was considered statistically significant.

## 3. Results

### 3.1. Protein Powder Administration Did Not Alter Body Weight, Kidney-to-Body Weight Ratio, or Conventional Renal Function Markers

To determine whether short-term administration of the different commercial protein powders affected general growth and conventional renal function, body weight, kidney-to-body weight ratio, serum urea and creatinine, and urinary parameters were evaluated after the 28-day experimental period. All 35 animals completed the 28-day administration period and no animal died.

Body weight was monitored throughout the study. At the end of the 28-day administration period, the mean body weight of the control group was 298 ± 14.8 g, and those of the whey, casein, soy, and beef protein groups were 306 ± 9.0 g, 305 ± 12.8 g, 316 ± 18.0 g, and 291 ± 20.2 g, respectively. Body weight gain did not differ significantly between the control group and any protein powder group (Figure 1A).

Kidney-to-body weight ratios were calculated to assess whether protein powder intake induced gross renal hypertrophy or atrophy. The mean kidney-to-body weight ratio was 0.7% in the control group and 0.6%, 0.7%, 0.6%, and 0.7% in the whey, casein, soy, and beef protein groups, respectively. These values did not differ significantly among the groups, suggesting that 28-day administration of protein powders did not alter relative kidney weight (Figure 1B).

Serum urea and creatinine were measured as conventional renal function markers. Serum creatinine levels were generally within or close to the reference range of 0.2–0.9 mg/dL (Figure 1C). The mean creatinine concentration was 0.52 ± 0.14 mg/dL in the control group, 0.70 ± 0.27 mg/dL in the whey group, 0.64 ± 0.08 mg/dL in the casein group, 0.59 ± 0.16 mg/dL in the soy group, and 0.66 ± 0.08 mg/dL in the beef group. Although the whey group showed a numerically higher mean creatinine level, this difference was not statistically significant (Figure 1C; *p* = 0.326). Urea levels remained within the reference range of 17–71 mg/dL in all groups, with mean concentrations of 39.43 ± 4.28 mg/dL (control), 35.43 ± 6.13 mg/dL (whey), 38.00 ± 3.46 mg/dL (casein), 39.71 ± 6.68 mg/dL (soy), and 43.00 ± 5.48 mg/dL (beef). No statistically significant difference was detected among the groups (Figure 1D; *p* = 0.142). Reagent-strip screening of urinary protein, pH and ketones detected no gross abnormality in any group.

Collectively, these findings indicate that 28-day administration of commercial protein powders did not induce overt renal functional impairment, as reflected by unchanged body weight, kidney-to-body weight ratio, serum urea and creatinine, and largely preserved urinary parameters. These conventional markers, and the oxidative stress parameters reported below, are secondary endpoints. As stated in Section 2.10, the study was powered for the primary histopathological outcome, and non-significant differences in the secondary endpoints should therefore be interpreted as inconclusive rather than as evidence of no effect.

### 3.2. Protein Powders Induced Source-Dependent Renal Histopathological Alterations

Although conventional renal function parameters were not significantly altered, histological examination revealed tubular alterations across all protein powder-treated groups. Kidney sections from the control group displayed normal renal architecture, with well-preserved cortical and medullary structures. The glomeruli, Bowman’s capsules, proximal and distal tubules, collecting ducts, and vascular structures all appeared intact, and no tubulointerstitial abnormalities, epithelial degeneration, or inflammatory changes were observed (Figure 2). In contrast, all protein powder-treated groups showed mild-to-moderate histopathological alterations relative to the control, including tubular dilation, epithelial desquamation, cytoplasmic vacuolization, and intertubular vascular congestion (Figure 2). Per-animal scores differed significantly among the groups for all four parameters (Kruskal–Wallis: tubular dilatation H = 17.01, *p* = 0.002; tubular desquamation H = 11.79, *p* = 0.019; tubular vacuolization H = 15.62, *p* = 0.004; intertubular vascular congestion H = 16.16, *p* = 0.003) and for the total renal injury score (H = 18.48, *p* = 0.001; Figure 3). Median total scores were 1 (range 1–4) in the control group and 6 (4–9), 6 (5–12), 7 (2–8) and 8 (5–10) in the whey, casein, soy and beef groups, respectively. The total score was significantly higher than control in the whey (*p* = 0.036), soy (*p* = 0.049) and beef (*p* = 0.0003) groups, and the casein group fell just short of significance (*p* = 0.085). The beef group was the only group that differed from control in all four parameters and showed the highest median total score; however, no comparison between protein powder groups reached significance for any parameter.

The whey, casein, and soy protein groups showed a broadly similar pattern of mild-to-moderate change, with comparable degrees of tubular dilation, focal epithelial desquamation, cytoplasmic vacuolization, and intertubular vascular congestion. In these three groups, glomerular structures were largely preserved, and no severe or degenerative tubular injury was observed. The beef protein group showed the most pronounced alterations, with the highest scores for tubular dilation and epithelial desquamation of all groups (Figure 2 and Figure 3).

These tissue-level changes occurred despite the absence of any significant change in conventional renal function markers.

### 3.3. Oxidative Stress and Antioxidant Enzyme Responses Differed According to Protein Source

We next asked whether these tissue-level changes were accompanied by an oxidative response, and measured renal MDA levels, CAT and GSH-Px activity, and plasma nitrite.

MDA, a marker of lipid peroxidation, differed significantly among the groups (one-way ANOVA, F(4,25) = 4.48, *p* = 0.007, η^2^ = 0.42). The beef protein group showed the highest MDA levels (3.54 ± 0.96 nmol/mg protein), significantly higher than the casein (2.43 ± 0.31; *p* = 0.030) and soy (2.39 ± 0.47; *p* = 0.010) groups. The difference from the control group (3.02 ± 0.20) did not reach significance. MDA levels in the whey group (2.67 ± 0.41) did not differ from those of any other group. These findings indicate that, among the protein sources tested, beef protein was associated with the highest renal lipid peroxidation (Figure 4A).

CAT activity also differed significantly among the groups (one-way ANOVA, F(4,29) = 7.65, *p* = 0.0002, η^2^ = 0.51). CAT activity was highest in the control group (0.287 ± 0.076 U/mg protein) and lowest in the casein group (0.115 ± 0.049). Activity was significantly lower than control in the casein (*p* = 0.0002) and whey (0.165 ± 0.038; *p* = 0.017) groups, and the casein group was also significantly lower than the beef group (0.240 ± 0.074; *p* = 0.009). The soy (0.207 ± 0.067) and beef groups did not differ from control. These findings indicate that antioxidant enzyme responses differed across protein sources, with casein administration associated with the most reduced CAT response (Figure 4C).

GSH-Px activity showed only small differences among the groups and did not differ significantly (one-way ANOVA, F(4,25) = 0.81, *p* = 0.532, η^2^ = 0.11). The soy protein group exhibited the highest mean GSH-Px activity and the casein group the lowest, while the whey, beef, and control groups were comparable. Because these differences were not statistically significant, the relatively higher GSH-Px activity in the soy group should be interpreted with caution (Figure 4B).

Plasma nitrite levels, used as an indirect indicator of nitric oxide metabolism, did not differ significantly among the groups (one-way ANOVA, F(4,29) = 0.74, *p* = 0.570, η^2^ = 0.09). Mean nitrite concentrations were lowest in the casein group and similar in the other groups, but no difference reached statistical significance (Figure 4D).

Taken together, the oxidative stress and antioxidant enzyme profiles showed protein source-dependent differences. Two distinct patterns emerged rather than a single gradient of severity. The beef protein group showed the highest MDA levels, significantly above the casein and soy groups, whereas the casein group showed the lowest CAT activity without a corresponding rise in MDA. GSH-Px and nitrite did not differ significantly among the groups. These findings suggest that the renal responses to different protein powders involve distinct oxidative stress-related patterns rather than a uniform mechanism across all sources.

### 3.4. Metal Exposure Estimates from Protein Powders Remained Below Toxicological Risk Thresholds

Renal histopathological and oxidative changes were present in all protein powder-treated groups. We therefore performed elemental analysis to establish whether the powders contained metal levels that could pose a toxicological concern under the applied intake scenario. The element concentrations detected in the protein powder samples are presented in Table 2. Because the analysis was conducted on the protein powders rather than on biological samples from the animals, this assessment was interpreted as an exposure-based toxicological risk evaluation rather than a direct comparison with the control group.

HRI values for the analyzed metals remained below 1 in every powder tested, indicating that the estimated daily metal intake did not exceed the accepted risk threshold. Similarly, estimated cadmium exposure remained below or within the TRV range, and lead exposure estimates remained below the IRL (Table 3).

These findings indicate that, under the present exposure assumptions, the tested protein powders did not contain heavy metal levels expected to pose a toxicological risk to kidney health. The renal histopathological alterations observed in the protein powder-treated groups are therefore unlikely to be primarily attributable to excessive heavy metal exposure from the tested products.

## 4. Discussion

This study evaluated the renal effects of 28-day oral administration of commercial protein powders from four sources (whey, casein, soy, and beef) in female Sprague Dawley rats. The principal finding was that, although conventional markers of renal function (serum urea and creatinine) remained unchanged in all groups, renal responses differed by protein source at the oxidative level. The beef group showed the highest MDA levels, and the whey and casein groups showed the largest reductions in CAT activity. Histological changes were present in all protein groups and were broadly similar in severity, with the beef group showing the highest injury scores and the clearest separation from control. These observations indicate that the renal impact of protein supplementation can be source-dependent even when standard functional markers remain normal [5]. It is important to distinguish two levels of these findings. At the structural level the four sources produced a broadly similar low-grade tubular response, with only the beef group standing apart and with differences too small to be resolved reliably by an ordinal scoring system. At the oxidative level, by contrast, the sources separated clearly and in a mechanistically interpretable direction. The claim of source dependence therefore rests primarily on the oxidative data, with the histological findings providing supporting rather than primary evidence.

The absence of significant changes in serum urea and creatinine indicates that none of the protein powders produced gross renal dysfunction over the 28-day period. This is consistent with meta-analytic evidence that higher protein intake does not adversely affect kidney function in healthy adults, in whom increased filtration appears to reflect a normal adaptive response rather than injury [5,6,7]. However, serum creatinine and urea are relatively insensitive to early or localized tubular injury because of renal functional reserve and the influence of non-renal factors such as muscle mass, hydration, and diet. In addition, a direct estimate of glomerular filtration rate from creatinine clearance was not available in the present study (see limitations below). Preserved serum markers therefore do not exclude subclinical, tissue-level changes, which is why histological and oxidative endpoints were included.

Histopathological examination showed that all protein powder groups developed mild-to-moderate tubular changes relative to the control, including tubular dilation, epithelial desquamation, vacuolization, and intertubular vascular congestion. The overall severity was broadly comparable across the four protein groups, with only modest differences among them; the beef group showed the most pronounced alterations, particularly tubular dilation and desquamation, whereas the whey, casein, and soy groups were closely similar. Given the semi-quantitative nature of the scoring and the limited separation among sources, these findings are best interpreted as a broadly similar low-grade tubular response to protein supplementation rather than a categorical difference among sources. This restrained interpretation is warranted because comparable ordinal scoring systems, although widely used to grade diet-related renal structural change in rats, are less sensitive than morphometric quantification [12].

The clearest evidence of source dependence came from the oxidative stress markers. The beef group showed the highest renal MDA levels, significantly above the casein and soy groups although not above the control. The whey and casein groups showed reduced CAT activity together with low MDA, and GSH-Px activity did not differ significantly among the groups. The elevated MDA in the beef group is mechanistically coherent: red meat proteins carry residual heme pigments (myoglobin and hemoglobin) that release iron during digestion. Heme iron is a well-characterized catalyst of lipid peroxidation through Fenton and Haber–Weiss chemistry, generating reactive aldehydes such as MDA [25,26]. Dietary heme iron has been shown to raise lipid-peroxidation biomarkers, including MDA, in rats [10]. Overall, these results indicate that renal oxidative responses to dietary protein are not uniform but reflect the biochemistry of the specific source. Two distinct patterns emerged rather than two degrees of the same one. The beef group showed a pro-oxidant profile, with the highest renal MDA concentrations among the sources tested. The casein group showed a different profile, in which MDA was not elevated, yet CAT activity was significantly reduced. This pattern is compatible with a low-intensity oxidative load that is gradually consuming the enzymatic defense rather than overwhelming it, and it may represent an earlier or slower process rather than a lesser one. The absence of pronounced casein histology indicates that this effect remained subclinical over the 28-day period. The description of beef as the source imposing the greatest oxidative burden therefore refers specifically to the magnitude of lipid peroxidation, not to the overall redox status of the tissue.

Two compositional properties may account for this source dependence. First, dietary acid load differs markedly between the sources tested. Animal proteins, and meat proteins in particular, are rich in the sulfur-containing amino acids methionine and cysteine, the oxidation of which yields sulfate and a net proton load, whereas soy protein carries a lower potential renal acid load [27]. Sustained acid loading increases renal ammoniagenesis, and intrarenal ammonia can activate the alternative complement pathway within the tubulointerstitium, a mechanism shown to drive tubulointerstitial injury in rats [28]. This is compatible with the pattern observed here, in which tubular dilation and epithelial desquamation were present in all protein groups and most pronounced after beef protein administration. Second, the sources differ in non-protein constituents with direct redox relevance. Whey is comparatively rich in cysteine, the rate-limiting precursor for glutathione synthesis. Casein is a phosphoprotein that delivers a higher phosphorus load, and high dietary phosphorus alone produces renal structural lesions in rats [12]. Soy protein carries isoflavones with documented anti-inflammatory and antioxidative renal effects [29]. These differences offer a plausible basis for the divergent antioxidant enzyme profiles observed, although the present design cannot isolate the responsible constituent, and studies using purified protein fractions would be required to do so.

The dose administered here (400 mg/kg/day of protein) is relatively low and lies within the range applied in previous 28-day repeated-dose rodent studies of protein supplementation, which reported no more than mild renal changes at comparable doses [13,14]. Notably, a source-dependent oxidative effect, most evident in the beef group, was detectable even at this modest dose, which corresponds to only a fraction of the intakes typical of habitual supplement users. Because of the interspecies difference in metabolic rate, this dose corresponds to approximately 65 mg/kg/day in humans [30], which is below the approximately 0.4 g/kg of protein provided by a single serving of a commercial protein powder in a 70 kg adult. This supports the interpretation that the source of protein, and not only the total amount consumed, can influence renal responses. Because only a single dose was tested, however, dose–response relationships could not be established, and it remains to be determined whether higher intakes or longer exposures would amplify these effects. Whey protein concentrate at 0.5 g/kg/day, only slightly above the dose used here, has been reported to induce hepatic oxidative stress in rats [31], underscoring the need for dose-ranging and chronic studies. The present findings can also be placed in a quantitative context. In a 28-day study of whey protein concentrate hydrolysate at 200, 400 and 800 mg/kg/day in Wistar-Kyoto rats, Hussein et al. reported no treatment-related change in serum urea or creatinine and normal renal histology at all doses [13]. In a 28-day study of a pea protein hydrolysate at 100, 200 and 400 mg/kg/day in Sprague Dawley rats, Hidayat et al. found serum urea and creatinine at 400 mg/kg/day comparable to control. Mild hyaline casts (score 1) were the only renal histological finding, and the no-observed-adverse-effect level was set at 200 mg/kg/day [14]. This combination of preserved serum markers with minor tubular change at the same dose as used here closely parallels our observations. For oxidative endpoints, Żebrowska-Gamdzyk et al. reported that whey protein concentrate raised hepatic MDA significantly at 0.5 g/kg/day but not at 0.3 g/kg/day over 7 to 21 days [31]. This threshold brackets the 400 mg/kg/day used here and is consistent with the absence of any rise in renal MDA in our whey group. Against this background, the beef group showed higher MDA than the casein and soy groups, and the whey and casein groups showed reduced CAT activity. Together, these findings indicate that the sources tested differ in their renal oxidative effects at a dose at which serum markers remain unchanged.

Elemental analysis indicated that the estimated exposures to nephrotoxic metals such as cadmium and lead remained below the reference values used for risk interpretation, with all HRI values below 1. Although cadmium and lead are established nephrotoxicants under sufficient or chronic exposure, the changes observed here are unlikely to be primarily attributable to the measured metal content of the powders. Because the elemental analysis was performed on the powders rather than on kidney tissue, tissue accumulation cannot be excluded, and future studies should incorporate tissue-level metal quantification.

This study has several limitations. First, only a single, relatively low dose was tested, so dose–response relationships could not be established; higher doses and longer-term exposure should be examined to determine whether the observed source-dependent effects are amplified or progress to functional impairment. Second, the 28-day exposure may not capture the cumulative effects of prolonged protein supplementation. Third, only female rats were studied and the stage of the estrous cycle was not determined at sacrifice. Females were selected deliberately: male rodents carry greater muscle mass and higher baseline serum creatinine, which confounds the interpretation of conventional renal markers, and female animals remain under-represented in the toxicological literature on protein supplementation. Cyclical variation in circulating estradiol nevertheless modulates both renal hemodynamics and antioxidant enzyme expression and may have increased within-group variance. Stratified randomization by baseline body weight distributed any such variation across groups at random, but cycle-staged or ovariectomized designs, and the inclusion of male animals, will be required before these findings can be generalized. Fourth, the protein powders were commercial products used as sold, and their protein identity and purity were based on manufacturer label information rather than independent analytical characterization, which may confound source-specific interpretation. Fifth, the oxidative and inflammatory assessment was limited to MDA, CAT, GSH-Px, and plasma nitrite, without broader redox markers such as the reduced-to-oxidized glutathione (GSH/GSSG) ratio, superoxide dismutase, protein carbonyls, or 8-hydroxy-2′-deoxyguanosine (8-OHdG), or dedicated inflammatory mediators. These markers are planned for future studies. Sixth, urinary creatinine was not measured, so glomerular filtration rate could not be estimated from creatinine clearance, and tissue-level metal accumulation was not determined; both are limitations of the present study. Creatinine clearance, the urinary protein-to-creatinine ratio, tubular injury markers such as KIM-1 and NGAL, and kidney metal quantification are planned for future studies. Seventh, renal injury was graded semi-quantitatively and morphometric quantification, such as tubular diameter and the area of cytoplasmic vacuolization, was not performed; these measurements are planned for future studies. The mechanistic interpretations offered here are therefore inferential and should be confirmed in dedicated studies using purified protein fractions and pathway-specific readouts.

From a translational perspective, healthy individuals consuming protein supplements within typical ranges are unlikely to develop overt renal dysfunction [5]. Nevertheless, the subclinical oxidative and tissue-level changes observed despite preserved serum creatinine caution against extrapolating “no functional change” to long-term safety, particularly for heavy users whose habitual intakes substantially exceed recommended levels. In such individuals, more sensitive tubular markers may be more informative than serum creatinine alone. The greater lipid peroxidation associated with beef protein is directionally consistent with current guidance favoring plant-predominant protein sources in adults with CKD at risk of progression, which recommends avoiding protein intakes above 1.3 g/kg/day [8]. However, the present study was conducted in healthy animals and cannot be extrapolated to a diseased kidney.

## 5. Conclusions

In conclusion, 28-day administration of commercial protein powders did not cause overt renal dysfunction, as conventional serum markers remained unchanged in all groups. However, all protein powders produced mild-to-moderate renal histological changes relative to the control, and the oxidative response was source-dependent. The beef group showed the highest MDA levels and the highest histological injury scores, whereas the casein group showed the largest reduction in CAT activity without a corresponding rise in lipid peroxidation. These are two different oxidative signatures rather than two degrees of the same one. These changes were unlikely to be driven primarily by heavy metal exposure, as the estimated metal intake remained below toxicological reference values. Taken together, the findings indicate that the renal effects of protein powders can be source-dependent even at a relatively low dose and in the absence of changes in conventional renal function markers. Among the sources tested, beef showed the highest lipid peroxidation. This highlights the need for renal safety assessment beyond conventional biochemical markers and warrants further evaluation of higher doses and longer-term exposure. From a practical standpoint, these findings do not indicate a renal hazard for individuals consuming protein supplements within recommended amounts. However, they suggest that habitual heavy users, particularly those relying on beef-derived products or with reduced renal reserve, may be better assessed with markers more sensitive than serum creatinine.

## Figures and Tables

**Figure 1:**
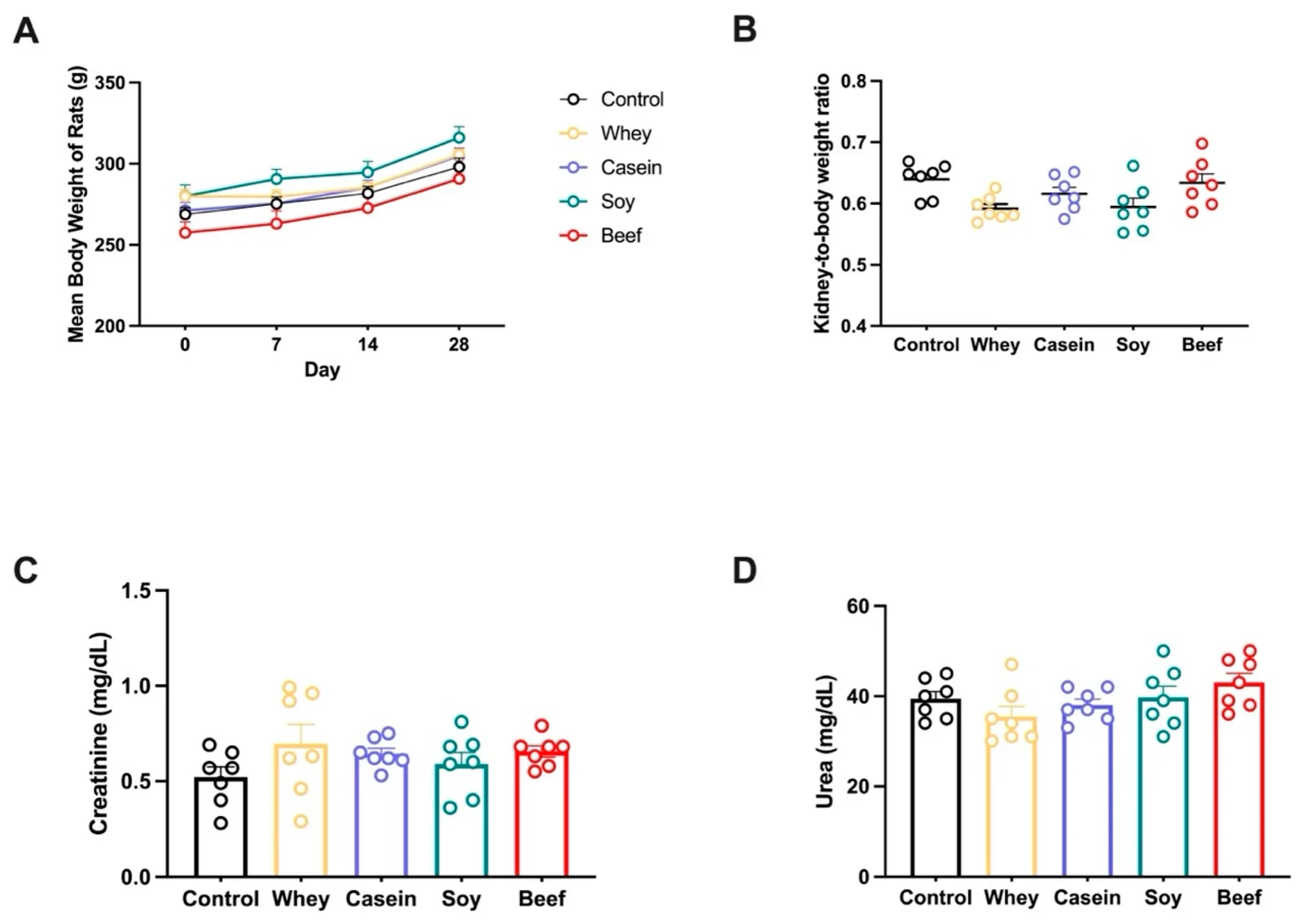
Body weight and conventional renal markers after 28 days of protein powder supplementation in female rats. (**A**) Body weight at days 0, 7, 14 and 28; (**B**) kidney-to-body weight ratio; (**C**) serum creatinine and (**D**) serum urea at day 28. Data are mean ± SD, *n* = 7 per group; no significant differences among groups (*p* > 0.05).

**Figure 2:**
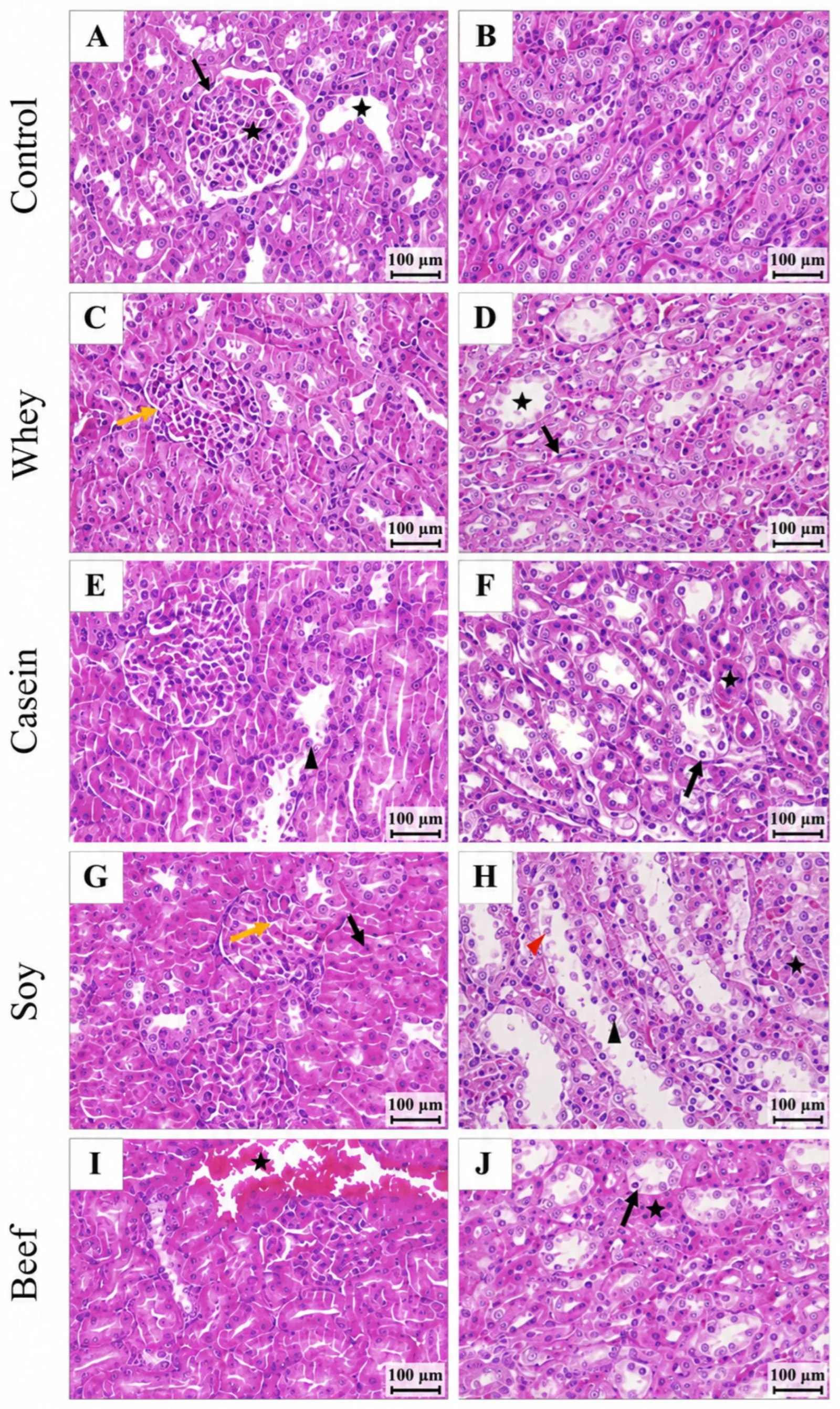
Representative hematoxylin and eosin-stained kidney sections after 28 days of protein powder supplementation in female rats. (**A**,**B**) control; (**C**,**D**) whey; (**E**,**F**) casein; (**G**,**H**) soy; (**I**,**J**) beef. Symbols: ★, glomerulus in (**A**), dilated tubular lumen in (**D**,**F**,**H**,**J**) and erythrocyte extravasation in (**I**); orange arrow, preserved glomerulus in (**C**,**G**); black arrow, renal tubule in (**A**) and tubular epithelial desquamation in (**D**,**F**,**G**,**J**); ▲, tubular dilation in (**E**,**H**); red arrowhead, desquamated epithelial cell within the tubular lumen in (**H**). Scale bars = 100 µm.

**Figure 3:**
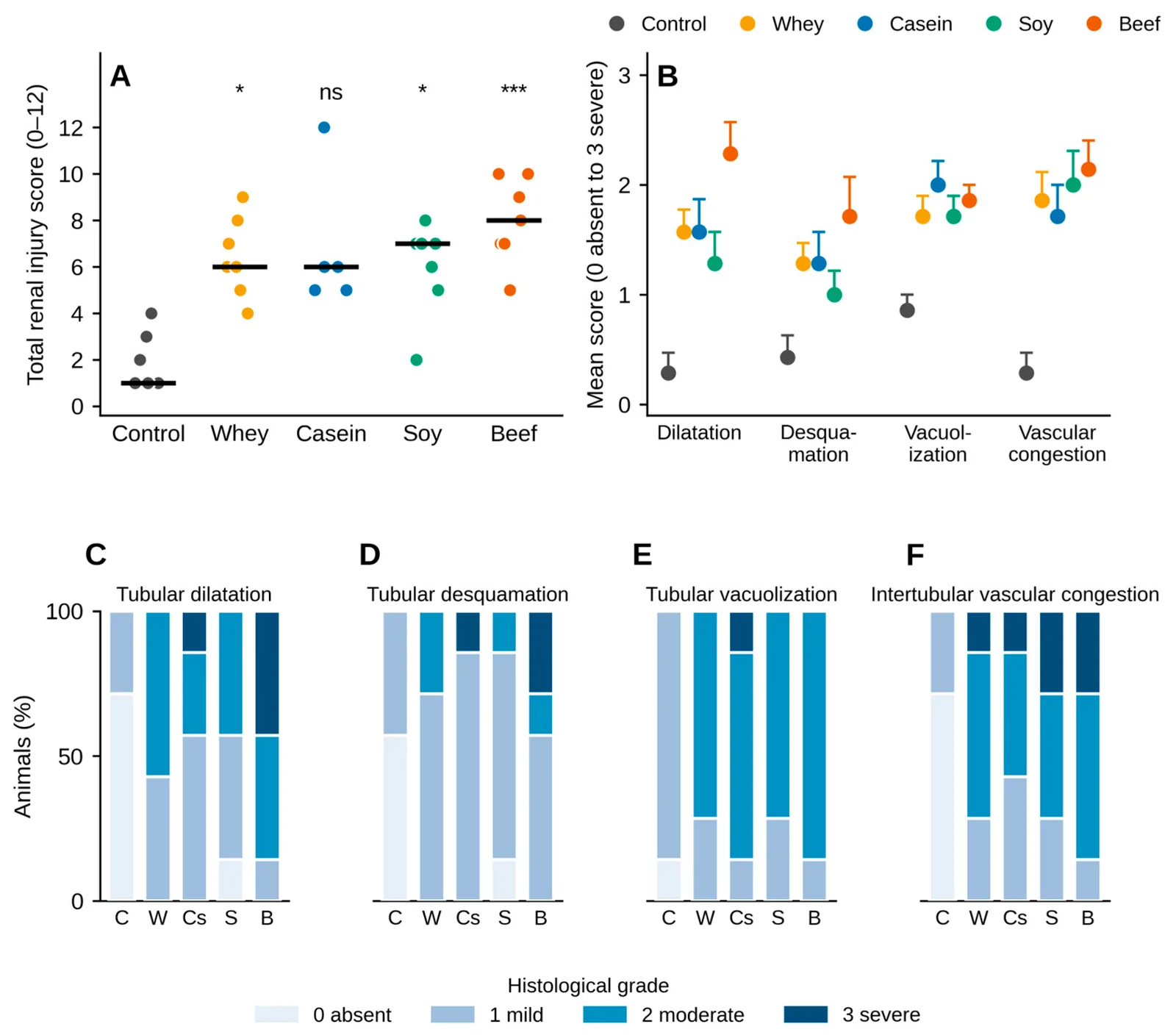
Semi-quantitative histopathological scores after 28 days of protein powder administration. (**A**) Total renal injury score (sum of the four parameters, range 0–12) for each animal; horizontal lines indicate the group median. Asterisks denote differences from the control group (Dunn’s post hoc test with Bonferroni correction after Kruskal–Wallis, H = 18.48, *p* = 0.001): * *p* < 0.05, *** *p* < 0.001, ns not significant. (**B**) Mean score (+ SEM) of each parameter by group. (**C**–**F**) Distribution of ordinal grades (0 absent, 1 mild, 2 moderate, 3 severe) for tubular dilatation, tubular desquamation, tubular vacuolization and intertubular vascular congestion. C, control; W, whey; Cs, casein; S, soy; B, beef; *n* = 7 per group.

**Figure 4:**
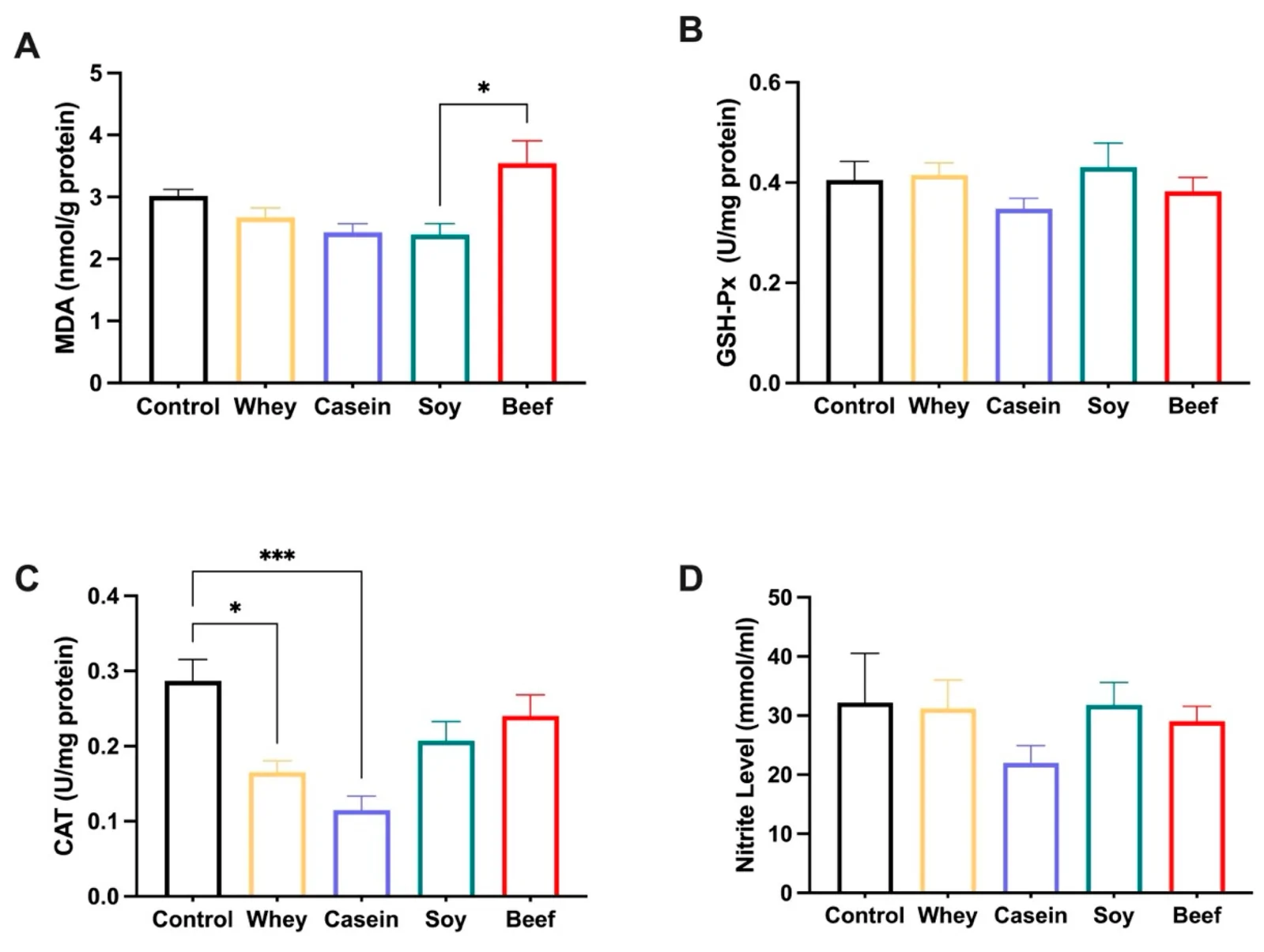
Oxidative stress parameters and nitrite levels following 28-day protein powder supplementation in female rats. (**A**) Malondialdehyde (MDA) concentrations, (**B**) Glutathione peroxidase (GSH-Px) activity, (**C**) Catalase (CAT) activity measured in kidney tissue homogenates and normalized to protein content determined by the Lowry method, and (**D**) nitrite levels measured in plasma. Data are presented as mean ± SD (*n* = 7 per group). Statistically significant differences between groups are indicated by * *p* < 0.05, *** *p* < 0.001 (one-way ANOVA with Bonferroni post hoc test).

**Table 1:** Declared nutritional composition of the protein powders administered to the rats.

Protein Powder	Serving (g)	Protein per Serving (g)	Fat (g)	Carb (g)	Energy (kcal)
Whey	30	24.07	1.2	6.3	120.17
Casein	30	24	1.2	0.7	113
Soy	25	21.95	0	0.475	90
Beef	35	24	1.2	0.6	137

Serving size, protein, fat, carbohydrate content, and energy per serving of whey, casein, soy, and beef protein powders. Protein density, calculated from the declared values, was 80.2% (whey), 80.0% (casein), 87.8% (soy) and 68.6% (beef) by weight. The administered dose was matched on actual protein content rather than on powder mass, so the mass of powder given differed between groups.

**Table 2:** Elemental composition of protein powders.

	Iron (μg/g)	Cadmium (μg/g)	Lead (μg/g)	Zinc (μg/g)	Copper (μg/g)
Whey	56.83	0.012	0.597	13.67	2.945
Casein	25.24	0.040	0.580	158.63	2.759
Soy	267.55	0.059	0.674	77.46	21.593
Beef	37.68	0.020	0.659	11.97	2.478

Iron, cadmium, lead, zinc, and copper concentrations were measured in each protein powder using ICP-MS and expressed in μg/g. Values reflect measured content for whey, casein, soy, and beef protein powders used in the study.

**Table 3:** Toxicological risk assessment of elements for protein powders.

	Iron	Cadmium	Lead	Zinc	Copper
**Whey protein**					
**HRI**	0.0316	-	-	0.0228	0.0368
**TRV**	-	0.0043	-	-	-
**IRL**	-	-	0.2985	-	-
**Safety**	Safe	Safe	Safe	Safe	Safe
**Casein**					
**HRI**	0.0351	-	-	0.2644	0.0345
**TRV**	-	0.0143	-	-	-
**IRL**	-	-	0.2900	-	-
**Safety**	Safe	Safe	Safe	Safe	Safe
**Soy**					
**HRI**	0.3716	-	-	0.1291	0.2699
**TRV**	-	0.0212	-	-	-
**IRL**	-	-	0.3368	-	-
**Safety**	Safe	Safe	Safe	Safe	Safe
**Beef protein**					
**HRI**	0.0523	-	-	0.0199	0.0310
**TRV**	-	0.0071	-	-	-
**IRL**	-	-	0.3297	-	-
**Safety**	Safe	Safe	Safe	Safe	Safe

Risk parameters: HRI values below 1 are considered safe. For cadmium, TRVs of 0.21–0.36 µg/kg body weight per day represent the tolerable range and values below 0.21 µg/kg body weight per day are considered safe (EFSA tolerable weekly intake, 2.5 µg/kg body weight [22]). For lead, IRL values below 8.8 µg/day, the FDA interim reference level for females of childbearing age [23], are considered safe. All risk levels were calculated for a daily intake of 25 g of protein powder, corresponding to a typical single serving of the products tested (declared servings 25–35 g; Table 1).

## Data Availability

The data presented in this study are available on request from the corresponding author.

## References

[1] Morton R.W., Murphy K.T., McKellar S.R., Schoenfeld B.J., Henselmans M., Helms E., Aragon A.A., Devries M.C., Banfield L., Krieger J.W. (2018). A systematic review, meta-analysis and meta-regression of the effect of protein supplementation on resistance training-induced gains in muscle mass and strength in healthy adults. Br. J. Sports Med..

[2] Detzel C., Petschow B.W., Johnson N., Weaver E.M. (2016). Comparison of the amino acid and peptide composition and postprandial response of beef, chicken, and whey protein nutritional preparations. Funct. Foods Health Dis..

[3] Valenzuela P.L., Mata F., Morales J.S., Castillo-García A., Lucia A. (2019). Does beef protein supplementation improve body composition and exercise performance? A systematic review and meta-analysis of randomized controlled trials. Nutrients.

[4] Kalantar-Zadeh K., Fouque D. (2017). Nutritional management of chronic kidney disease. N. Engl. J. Med..

[5] Devries M.C., Sithamparapillai A., Brimble K.S., Banfield L., Morton R.W., Phillips S.M. (2018). Changes in kidney function do not differ between healthy adults consuming higher- compared with lower- or normal-protein diets: A systematic review and meta-analysis. J. Nutr..

[6] Van Elswyk M.E., Weatherford C.A., McNeill S.H. (2018). A systematic review of renal health in healthy individuals associated with protein intake above the US Recommended Daily Allowance in randomized controlled trials and observational studies. Adv. Nutr..

[7] Antonio J., Ellerbroek A., Silver T., Vargas L., Tamayo A., Buehn R., Peacock C.A. (2016). A High Protein Diet Has No Harmful Effects: A One-Year Crossover Study in Resistance-Trained Males. J. Nutr. Metab..

[8] Stevens P.E., Ahmed S.B., Carrero J.J., Foster B., Francis A., Hall R.K., Herrington W.G., Hill G., Inker L.A., Kazancıoğlu R. (2024). KDIGO 2024 clinical practice guideline for the evaluation and management of chronic kidney disease. Kidney Int..

[9] Ogborn M.R., Bankovic-Calic N., Shoesmith C., Buist R., Peeling J. (1998). Soy protein modification of rat polycystic kidney disease. Am. J. Physiol. Ren. Physiol..

[10] Bastide N.M., Chenni F., Audebert M., Santarelli R.L., Taché S., Naud N., Baradat M., Jouanin I., Surya R., Hobbs D.A. (2015). A central role for heme iron in colon carcinogenesis associated with red meat intake. Cancer Res..

[11] Zhang W.R., Parikh C.R. (2019). Biomarkers of acute and chronic kidney disease. Annu. Rev. Physiol..

[12] Esquinas P., Rios R., Raya A.I., Pineda C., Rodriguez M., Aguilera-Tejero E., Lopez I. (2021). Structural and ultrastructural renal lesions in rats fed high-fat and high-phosphorus diets. Clin. Kidney J..

[13] Hussein F.A., Chay S.Y., Ghanisma S.B.M., Zarei M., Auwal S.M., Hamid A.A., Wan Ibadullah W.Z., Saari N. (2020). Toxicity study and blood pressure–lowering efficacy of whey protein concentrate hydrolysate in rat models, plus peptide characterization. J. Dairy Sci..

[14] Hidayat M., Prahastuti S., Rakasiwi A.S., Prisilia S., Hasan K. (2022). Sub-chronic toxicity study of green peas protein hydrolysate in rats. Toxicol. Rep..

[15] Percie du Sert N., Hurst V., Ahluwalia A., Alam S., Avey M.T., Baker M., Browne W.J., Clark A., Cuthill I.C., Dirnagl U. (2020). The ARRIVE guidelines 2.0: Updated guidelines for reporting animal research. PLoS Biol..

[16] Lowry O.H., Rosebrough N.J., Farr A.L., Randall R.J. (1951). Protein measurement with the Folin phenol reagent. J. Biol. Chem..

[17] Ohkawa H., Ohishi N., Yagi K. (1979). Assay for lipid peroxides in animal tissues by thiobarbituric acid reaction. Anal. Biochem..

[18] Aebi H. (1984). Catalase in vitro. Methods Enzymol..

[19] Paglia D.E., Valentine W.N. (1967). Studies on the quantitative and qualitative characterization of erythrocyte glutathione peroxidase. J. Lab. Clin. Med..

[20] Green L.C., Wagner D.A., Glogowski J., Skipper P.L., Wishnok J.S., Tannenbaum S.R. (1982). Analysis of nitrate, nitrite, and [15N]nitrate in biological fluids. Anal. Biochem..

[21] Cui Y.-J., Zhu Y.-G., Zhai R.-H., Chen D.-Y., Huang Y.-Z., Qiu Y., Liang J.-Z. (2004). Transfer of metals from soil to vegetables in an area near a smelter in Nanning, China. Environ. Int..

[22] (2011). EFSA Panel on Contaminants in the Food Chain (CONTAM). Statement on tolerable weekly intake for cadmium. EFSA J..

[23] Flannery B.M., Middleton K.B. (2022). Updated interim reference levels for dietary lead to support FDA’s Closer to Zero action plan. Regul. Toxicol. Pharmacol..

[24] Faul F., Erdfelder E., Lang A.G., Buchner A. (2007). G*Power 3: A flexible statistical power analysis program for the social, behavioral, and biomedical sciences. Behav. Res. Methods.

[25] Carlsen C.U., Møller J.K.S., Skibsted L.H. (2005). Heme-iron in lipid oxidation. Coord. Chem. Rev..

[26] Papuc C., Goran G.V., Predescu C.N., Nicorescu V., Stefan G. (2017). Mechanisms of oxidative processes in meat and toxicity induced by postprandial degradation products: A review. Compr. Rev. Food Sci. Food Saf..

[27] Remer T., Manz F. (1995). Potential renal acid load of foods and its influence on urine pH. J. Am. Diet. Assoc..

[28] Nath K.A., Hostetter M.K., Hostetter T.H. (1985). Pathophysiology of chronic tubulo-interstitial disease in rats. Interactions of dietary acid load, ammonia, and complement component C3. J. Clin. Investig..

[29] Jheng H.-F., Hayashi K., Matsumura Y., Kawada T., Seno S., Matsuda H., Inoue K., Nomura W., Takahashi H., Goto T. (2020). Anti-inflammatory and antioxidative properties of isoflavones provide renal protective effects distinct from those of dietary soy proteins against diabetic nephropathy. Mol. Nutr. Food Res..

[30] Reagan-Shaw S., Nihal M., Ahmad N. (2008). Dose translation from animal to human studies revisited. FASEB J..

[31] Żebrowska-Gamdzyk M., Maciejczyk M., Zalewska A., Guzińska-Ustymowicz K., Tokajuk A., Car H. (2018). Whey protein concentrate WPC-80 intensifies glycoconjugate catabolism and induces oxidative stress in the liver of rats. Nutrients.

